# The incidence, clinicopathological characteristics and the survival outcomes among young breast cancer patients in Alexandria, Egypt

**DOI:** 10.3332/ecancer.2026.2074

**Published:** 2026-02-06

**Authors:** Abeid Omar, Omar Shebl, Abdelaziz Belal, Azza Darwish

**Affiliations:** 1 Department of Clinical Oncology and Nuclear Medicine, Faculty of Medicine, 21526 Alexandria University, Alexandria, Egypt; 2 Department of Clinical Oncology and Nuclear Medicine, Kenyatta University Teaching, Referral and Research Hospital, 00100 Nairobi, Kenya

**Keywords:** breast cancer, developing countries, low and middle income countries, overall survival, disease-free survival, incidence

## Abstract

Breast cancer (BC) is predominantly considered a postmenopausal disorder in Western countries, with only 6% being in women below 40. In contrast, developing low- and middle-income countries (LMIC) see a much higher incidence of BC in premenopausal women due to genetic and demographic factors—reaching as much as 25%. The lack of well-established data on the features and prevalence of BC in premenopausal females in LMIC calls for a wholesome assessment of its features. Our retrospective study was conducted at two major oncology centers in Alexandria, Egypt. We included patients aged 18–40 years diagnosed from January 2008 to December 2017. We included non-metastatic patients and extracted data from files and charts. We assessed patients for clinicopathological characteristics, treatments and survival outcomes. Statistical analysis was performed using SPSS Version 2.0, and survival was assessed by Kaplan–Meir curves. Results were considered statistically significant at a *p*-value <0.05. After a median follow-up of 41 months, more than one-third of patients experienced relapse, with more cases of distant recurrence (73%) than locoregional recurrence (27%). The median disease-free survival (DFS) was 78 months, and the 5-year overall survival was 92%, both of which were independently predicted by tumour size and nodal status for DFS only. Our cohort had comparable features compared to prior studies conducted in Europe and the States. We also found some similarities to Kenya regarding BC in the young, where one study done in Kijabe, Kenya, showed a median presentation age of 38.2 years and a DFS of 75 months.

## Background

In 2022, it was estimated that one out of eight women would be diagnosed with breast cancer (BC), with an estimated over 2.3 million new BC cases, making it the second most common cancer globally, with the majority of new cases occurring in transitional countries, that is, low and middle-income countries (LMIC) [1].

In Western countries, BC predominantly occurs in postmenopausal women, with about 6% diagnosed in women 40 years and below. Contrastingly, in developing countries, Latin and Asian countries, BC is diagnosed frequently in premenopausal women, with about 25% being below the age of 40 years [2–4].

The high incidence of BC in developing countries, particularly the young women, is attributed to the change of lifestyle to that of the Western type, such as delayed first pregnancy, change in diet to that rich in fats and carbs, among others [4].

It is well established in the western world that compared to BC occurring in older women, BC in young women is associated with poor prognosis [5]. This is considered due to the aggressive biology of BC in young women characterised by high incidence of lymphovascular involvement (LVI), high tumour grade, more lymph node involvement, larger tumour size, less hormone receptors expression, more triple negative breast cancer (TNBC) and human epidermal growth factor receptor 2 (HER2) BC subtype [6]. The larger tumour size and high positive lymph nodes could be due to the lack of screening of this age group and poor health-seeking behaviour compared to the older population group. In addition, young women have dense breast, which might make it difficult to notice breast lumps [7]. Moreover, young women have been shown to have poorer compliance to endocrine therapy (ET) due to the side effects and the facts some of them may want to become pregnant to complete their families.

When it comes to treatment, BC in young has been treated more aggressively. Frequently, young women undergo more mastectomies than breast conserving surgeries (BCSs) [8]. They also frequently treated with ovarian function suppression (OFS). However, most of the guidelines are against the use of aggressive treatment of BC in young solely due to age. Instead, treatment should be guided by biology [9].

Currently, there is no consensus in the definition of BC in young, the European School of Oncology; European Society for Medical Oncology panel defines BC in young as that occurring in the age less than 40, while the very young are defined as those less 35 years [9]. The American Society of Clinical Oncology has defined Adolescent and Young Adult (AYA) to mean BC in young that which occurs between 15 and 39 years of age, while in many clinical trials, such as SOFT and TEXT trials, the premenopausal women or those less than 50 years of age are considered as young [10, 11]. For the purpose of this work, we will define young women to be those 40 years and below, while very young as those below 35 years.

The recently published GLOBOCAN data has shown the global burden of BC will be due to LMIC and most of the patients will be young [1]. While there is well-established data of BC in young in the West, there is a scarcity of data from the LMIC, yet the burden of BC in young occurs in these countries. Thus, there is an unmet need to describe the clinical characteristics of BC in young in LMIC, define the treatment used and their outcome. We hereby present one of the largest BC types in young women with 10-year data from two institutions in Alexandria, Egypt.

## Methods

This retrospective study was conducted in two centers in Alexandria in Egypt: Ayadi Almostakbal Cancer Center and Alexandria University Hospital. It included patients aged 18–40 diagnosed between January 2008 and December 2017. We included patients who were non-metastatic (stages I–III), and metastatic patients were counted for the purpose of incidence.

We extracted data from the patients’ files and charts. Data extracted included: their age, parity, the use of contraceptives, family history of BC, onset of breast changes till seeking medical attention, the tumour size, nodal status, the Tumor, nodes, and metastasis (TNM) staging according to The 8^th^American Joint Committee on Cancer the status of the estrogen receptors (ERs), progesterone receptors (PRs), HER2 and fluorescence *in situ* hybridisation where necessary, the type of surgery performed (BCS versus mastectomy), type of chemotherapy given (taxanes, anthracyclines and so on), OFS, radiotherapy, trastuzumab usage and type of ET.

We aimed to find the incidence, clinicopathological characteristics, treatment used, disease-free survival (DFS) and overall survival (OS) of BC in young. Incidence was defined as the percentage of women with BC aged 40 and below from the total BC patients. The DFS was determined from the day of confirmed histological diagnosis to the first recurrence or death whichever occurred first, while the OS was from the time of diagnosis until lost to follow-up or for the patients who were still alive until the time of cutoff data in December, 2019.

The data analysis was conducted with SPSS version 2.0. Descriptive statistics was conducted. Survival was assessed by Kaplan–Meir curves. Univariate analysis was based on Kaplan–Meier curves. Cox regression was used for multivariate analysis, and results were considered statistically significant at a *p*-value <0.05 was considered significant.

This study had the ethical approval of the Faculty of Medicine of Alexandria University.

## Results

**Figure 1.**
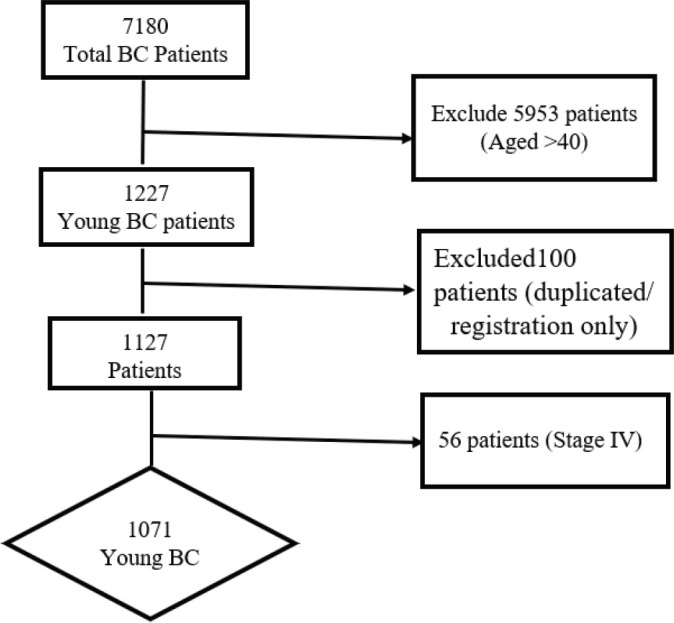
The flowchart of patients’ recruitment. BC: Breast cancer.

Between 2008 and 2017, a total of seven thousand, one hundred and eighty BC patients were registered in the two centers. Out of these, 17% (*n* = 1227) were aged ≤40 (BCY). Among the BCY, one hundred patients were either duplicated or only registered but never treated in either of the two hospitals, hence were excluded. In the remaining 1,127 patients, 56 patients presented with de novo stage IV and were further excluded. We focused our analysis on the BCY patients with stages I–III disease (*n* = 1071) as illustrated in Figure 1**. In the included patients, around 9% of the patients had some missing documentation data, varied from one variable to another, so these were excluded from the statistical analysis of each variable and the total number of included patients in each variable is mentioned in our data tables (**Tables 1–3**). In addition, a total of 12% of the patients were lost to follow-up at some point from 2008 to 2017.**

**Figure 2.**
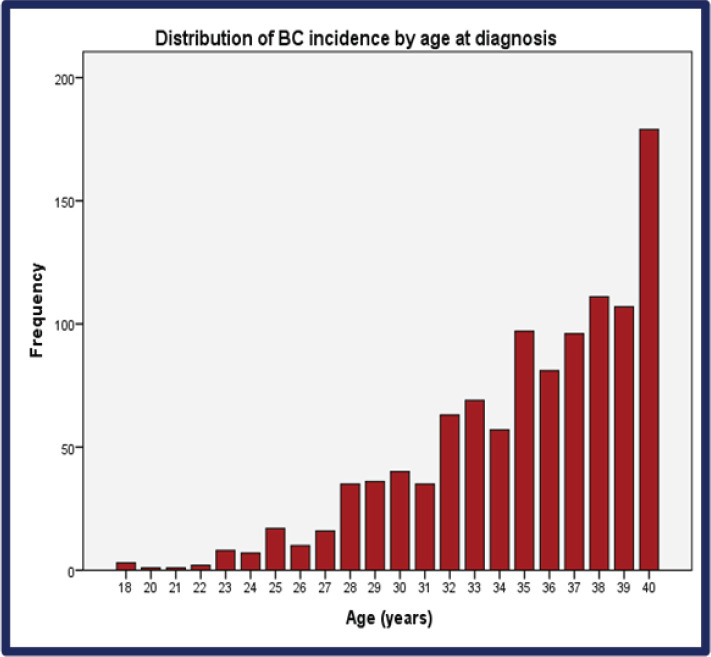
Distribution of BC incidence by age at diagnosis. BC: Breast cancer.

**Figure 3.**
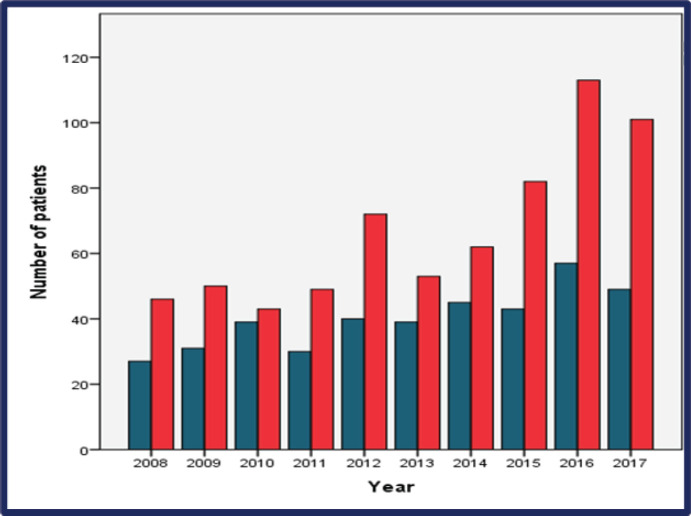
Distribution of the incidence of BC in young by age group (very young in blue versus young in red) per year of diagnosis.

From 2008 through 2017, the incidence of BCY increased steadily, as shown in Figure 2. Overall, the median age at diagnosis was 36 years (range: 18–40) (Figure 2** and** Table 1). Of which 37.3% were very young (<35 years) (Figure 3). Only seventeen percent (*n* = 129) of the patients had a positive family history of BC. Sixty percent (*n* = 443) of the total patients were obese. The median time from the onset of breast changes to presenting to the healthcare provider was 3 months. The majority (58%, *n* = 386) of the patients presented ≥90 days (presentation delay) from the onset of breast changes to presenting to the hospital Table 1**.**

The pathological characteristics are summarised in Table 2**.** In terms of tumour size majority had T1 and T2 disease (24% and 56%, respectively). Only a third of the patients had negative lymph node metastasis at diagnosis. In the overall TNM stages, stages II and III were very frequent, 44% each. The most common histological subtype was invasive ductal carcinoma (IDC) (87.9%, *n* = 904), while classical invasive lobular carcinoma (ILC) was rarer (2.9%). Most patients had intermediate to high-grade tumours (77.8% and 19.6%, respectively). Ki-67 was not routinely performed, but among the patients in which it was done majority of them (62%, *n* = 97) had high Ki-67 (>20%). Forty percent of the patients had extracapsular extension (ECE) involvement. ER/PR positive was present in 80%, HER2 positive in 23% and TNBC in 12%.

The treatment used is summarised in Table 3. Mastectomy was the most commonly performed surgery (66%), with only a third of the patients undergoing BCS. Only 9% of the patients were treated by neoadjuvant chemotherapy, with the majority (84%) receiving chemotherapy in the adjuvant set-up, while 7% were treated with both neoadjuvant and adjuvant chemotherapy. The most commonly used chemotherapy regimen was either anthracycline-based (60%) or combined anthracycline and taxanes (37%). Hypofractionated radiotherapy was utilised more frequently (47%) than conventional radiotherapy (34%). Out of the one hundred and nineteen HER2 positive patients, only 63% were confirmed to have received trastuzamab. However, 37% had missing data on whether they were treated with trastuzumab. The majority of the hormone receptor-positive patients were treated with tamoxifen (data not shown). Only 9% of the patients had OFS used, with 14% experiencing amenorhoea. Amenorhoea was assessed by the absence of monthly menstruation period from the initial use of chemotherapy as estradiol levels were not frequently measured.

After a median follow-up of 41 months (interquartile range – IQR: 34–63), 36.4% of the patients relapsed during this period. Out of these 347 patients, 253 (73%) patients developed distant relapse and 94 developed local recurrence (27%).

**Figure 4.**
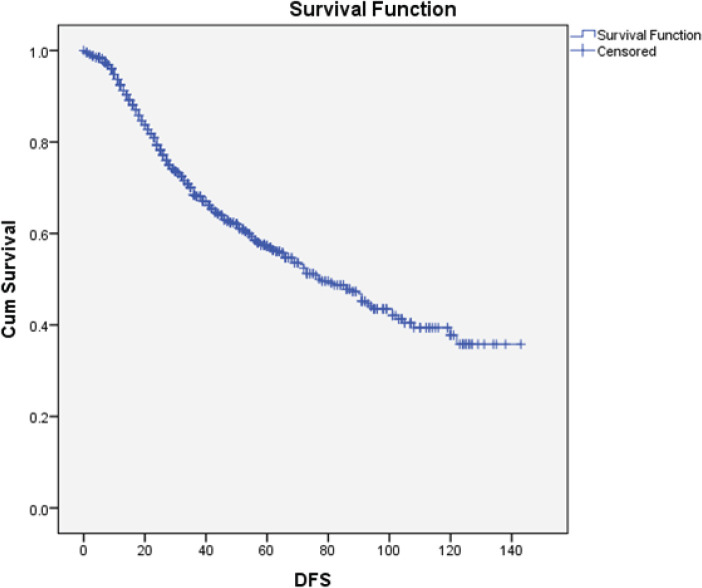
Kaplan-Meier curve for DFS. DFS: Disease-free survival; Cum Survival: Cumulative survival.

The median DFS was 78 months (95% CI: 66–89). After adjusting for confounders by Cox regression, only the tumour size and *N*-status were independent predictors for DFS (Figure 4).

**Figure 5.**
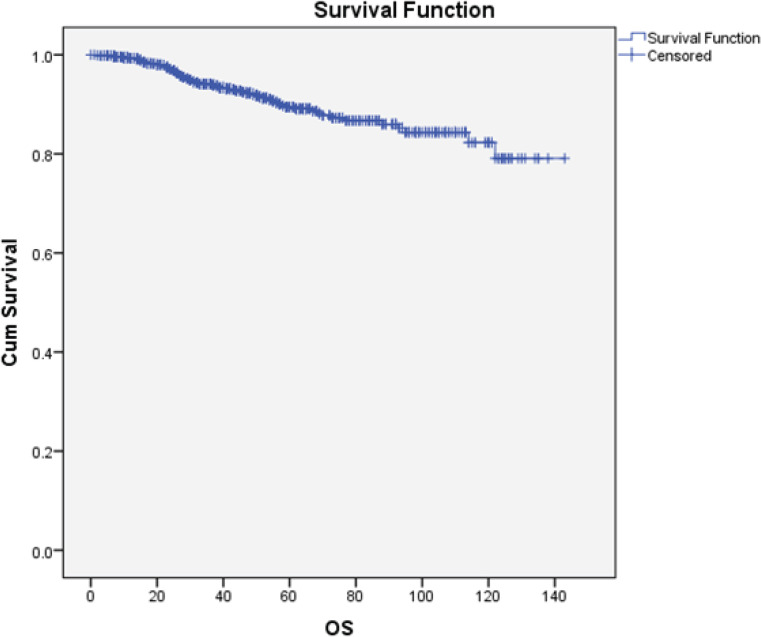
The Kaplan-Meier curves for OS. OS: Overall survival; Cum Survival: Cumulative survival.

The OS was 92%. The median OS was not reached, while the mean OS was 127 months (CI 95%: 123–130) Figure 5.

In predictors of OS, the tumour size, nodal status, type of surgery and ECE were significantly associated with OS. However, only *T*-size was an independent predictor of OS in multivariate analysis.

## Discussion

This study included young BC patients aged ≤40 years (BCY) diagnosed between 2008 and 2017 in two centers in Alexandria, Egypt. The incidence of BCY was 17% and increased gradually during the study period.

The global incidence of BC has been increasing steadily across all age groups, including those below 50 years old. The increase in incidence of BC in the young is particularly higher in the LMIC compared to that in the Western world [12]. In this study, the incidence of BCY increased from 2008 to 2017, reaching nearly 20% which is considerably high compared to that of Europe and the USA.

In LMIC countries, most women have a delay in presentation from the time they notice the breast changes [13, 14]. In Kenya, women present more than 17 weeks from the time they notice breast changes, with an average age of presentation of 50 years [13]. The majority of the patients in this study had delays in presentation. It took more than 3 months to seek medical assistance after they had noticed the breast changes, consequently most of the patients were diagnosed with large tumour size, more nodal involvement and overall higher TNM stage. Presentation delay has been associated with advanced disease at presentation leading to poorer survival [15]. It should be noted that, unlike older women, BC screening by mammogram is not routinely conducted in younger women due to denser breasts. Moreover, mammography imaging is not readily available in most LMICs [14]. Perhaps using clinical breast examination might improve the situation in these countries [16].

Previous studies have characterised BC in the young as having more aggressive features, such as a higher incidence of positive lymph nodes, large tumour size, more LVI and ECE involvement, less ER positivity, higher TNBC and HER2+ BC than the older counterparts [17]. Nevertheless, some studies have shown that there is a difference in clinicopathological characteristics among Asian women and those in Europe or the USA, particularly in terms of receptor expression [18]. In the review by Azim and Partridge [6], the frequency of hormone receptor-positive BC in young women was up to 63% in Western countries. In contrast, a study comparing the ER rates in young BC women in Asia and the USA showed that the young Asian women had higher ER positivity rates than their counterparts in the USA [19]. Among the Argentine, Jordan and Taiwan BC patients, the young patients were demonstrated to have an ER/PR positivity rate of 74%, which is almost comparable to our study population, 80% [17, 20, 21]. On the other hand, the incidence of

TNBC in our study was lower compared to that reported in the USA and Europe among young BC patients but similar to the Argentine women [6, 19, 21]. Similarly, prospective data from Kenya shows an ER positivity rate of 74% while TNBC being 20% [13]. In a study from the National Cancer Institute of Egypt, their cohorts had close rates of hormone receptor-positive patients as our study (71%) [22]. This highlights the genetic and environmental difference in BC among young patients in different parts of the world, something which warrants a global exploration [19, 20].

Young patients tend to undergo more aggressive treatments, such as more mastectomies than BCS compared to older patients [8]. In a study comparing the AYA and older women, Murphy *et al* [8] found that the young women were treated more aggressively. These women underwent more mastectomies than their older counterparts [8]. Similarly, in this study, mastectomies were more commonly performed than the BCS. The explanation could be due to higher TNM stages at presentation in both studies. Another possible reason could be young patients tend to have higher local recurrences if BCS is done than mastectomy, which could lead to a bias towards mastectomies [8]. A shared doctor-patient decision should be discussed on the type of surgery (based on disease stage and feasibility), as it has been shown that mastectomy is associated with anxiety and depression compared to BCS [23].

Despite most of our patients being ER-positive, nearly all of them were treated by chemotherapy: fewer women were treated in a neoadjuvant setup, and the most commonly used chemotherapy was anthracyclines with or without taxanes. Considering that most young patients have a high disease burden at diagnosis, neoadjuvant chemotherapy should be advocated as it may downsize the tumours and make BCS possible [24]. Although few young BC patients were included in the TAILORX trial [25], the 21-gene recurrence score still has a role in the young ER-positive patients [26]. Whenever feasible this should be offered to patients to avoid unnecessary chemotherapy side effects.

Young BC women have high recurrence rates both locoregional and distant metastasis. However, mostly the local recurrence is higher in women who have undergone BCS [27]. However, most data suggests that the local recurrence has no impact on OS, with few studies showing otherwise [28–30]. In the univariate analysis, the *T* size, Nodal Status, TNM stage, LVI, ECE and the type of surgery significantly contributed to relapses. However, only the *T*-size and *N*-positivity remained significant in the Cox regression similar as reported in other previous studies [21, 31]. Nevertheless neither did age, as a continuous variable, nor as a categorical one have an impact in survival outcomes [32].

One study performed in on Kenyan patients [33] has also reported similar outcomes to ours, yet with some differences in some respects. For example, the authors reported a similar median age of diagnosis, 38.2 years. In addition, out of the included Kenyan patients, 61% patients were luminal BC patients, while 21% were TNBC. The authors in the study reported a median DFS of 6.3 years (i.e., around 75 months) as well as relapse rates of 20% in younger patients.

Similarly, multiple other studies have previously tried to assess the present clinical characteristics of BC in the young in Egypt—though on a much smaller patient cohort. One of these studies that were conducted at Minia Oncology center [34] and it assessed the clinical characteristics of 100 young BC patients. The authors reported a luminal BC rate of 74%—a little bit lower than our study. The cohort in the studies had 17% stage I BC and 54% had stage III patients had stage III cancer. Another study conducted in Mansoura on 300 young patients (≤35 years) also tried to assess similar data [35]. The included cohort was mostly non-metastatic (95.3%), and they reported around 24% of N0 disease and 27% of N3 disease. In contrast to our findings, only 52.7% had luminal BC in that study, 20.8% had Her2-enriched and 27% had TNBC. Such molecular differences in the Mansoura Oncology Center can probably explain why the median DFS was lower compared to ours, 61 months only, as well as the lower 5-year OS, which was only 68%—compared to our 92%.

When comparing our findings to studies in both Kenya and other institutions in Egypt, we can see that there are inter-institutional differences even within the same country, and that molecular distribution is mostly the main driver for such differences. More larger studies on BC in the young may still be needed to better characterise this cohort of patients.

Our study had the limitations of being retrospective with some patients having some missing data, thus the results should be interpreted with caution. In addition, the lack of data, whether due to lack of documentation (around 9%) or loss to follow-up (around 12%), is another limitation. However, our findings still reflect what is in the literature. Moreover, this is one of the largest studies of BC in young in the Middle East and Sub-Saharan Africa.

## Conclusion

To conclude, BC in the young was associated with aggressive clinicopathological features, which led to more aggressive treatments and higher recurrence rates compared to postmenopausal females. However, our cohort still had good median DFS and OS rates, both of which were mostly dependent upon initial tumour size and nodal status.

## List of Abbreviations

AYA, Adolescent and young adult; BC, Breast cancer; BCS, Breast conserving surgery; DFS, Disease-free survival; ECE, Extracapsular extension; ER, Estrogen receptor; ET, Endocrine therapy; Her2, Human epidermal growth factor receptor 2; IDC, Invasive ductal carcinoma; ILC, Invasive lobular carcinoma; LVI, Lymphovascular invasion; LMIC, Low- and middle-income countries; OFS, Ovarian function suppression; OS, Overall survival; PR, Progesterone receptor; TNBC, Triple negative breast cancer.

## Conflicts of interest

We report no conflicts of interest for this meta-analysis.

## Funding

This research did not receive any specific grant from funding agencies in the public, commercial or not-for-profit sectors.

## Figures and Tables

**Table 1.** Clinical characteristics of the included patients.

Characteristics	Total *n* (%)
Distribution of the patients	1,071 (100%)
Age at diagnosis Median IQR Range	36 32–39 18–40
Body mass index Median Range IQR	31 16–72 27–36
WHO BMI (*n* = 702) Under weight Normal weight Overweight Obesity	12 (1.6%) 85 (11.5%) 196 (26.6%) 445 (60.3%)
Family history (*n* = 735) None First degree Second degree Not specified	576 (78.4%) 62 (8.4%) 67 (9.1%) 30 (4.1%)
Oral contraceptive use (*n* = 505) Yes No	136 (26.9%) 369 (73.1%)

n: number; WHO: World Health Organisation; BMI: Body mass index; IQR: Interquartile range

**Table 2.** Pathological characteristics of the included patients.

Characteristics	Overall *n* (%)
Histological subtype (*n* = 1,029) IDC ILC IDC+ILC Other	904 (87.9%) 30 (2.9%) 25 (2.4%) 70 (6.8%)
Tumour grade (*n* = 963) 1 2 3	25 (2.6%) 749 (77.8%) 189 (19.6%)
Ki-67 Low High	59 (37.8%) 97 (60.8%)
Focality (*n* = 1,041) Unifocal Multifocal	828 (79.5%) 21 (20.5%)
Tumour size (*n* = 963) T1 T2 T3 T4	233 (24.2%) 543 (56.4%) 149 (15.5%) 38 (3.9%)
Nodal status (*n* = 1,010) N0 N1 N2 N3	315 (31.2%) 293 (29.0%) 248 (24.6%) 154 (15.2%)
TNM Stage (*n* = 976) I II III	110 (11.3%) 429 (44.0%) 437 (44.8%)
Molecular subtype (*n* = 959) Luminal (ER/PR+, ±HER2+) HER2 Enriched (ER-/HER2+) TNBC (ER,PR, HER2 -ve)	770 (80.3%) 72 (7.5%) 117(12.2%)
ECE (*n* = 765) Neg Pos	456(59.6%) 309(40.4%)
LVI (*n* = 759) Negative Positive	289 (38.1%) 470 (61.9%)

n: number; IDC: Invasive ductal carcinoma; ILC: Invasive lobular carcinoma; T: Tumour size; N: Nodes; M: metastasis; ER: Estrogen receptor; PR: Progesterone receptor; Her2: human epidermal growth factor receptor 2; TNBC: Triple negative breast cancer; ECE: Extracapsular extension; LVI: Lymphovascular invasion

**Table 3.** Types of therapy received.

Characteristics	Overall *n* (%)
Surgery (*n* = 1,015) Mastectomy BCS	674 (66.4%) 341 (33.6%)
Chemotherapy type (*n* = 978) Anthracycline based Taxane based Combined Not specified	584 (59.7%) 5 (0.5%) 364 (37.2%) 25 (2.6)
Chemotherapy timing (*n* = 979) Adjuvant only Neoadjuvant only Both	826 (84.4%) 87 (8.9%) 66 (6.7%)
Radiotherapy Conventional Hypofractionated Unspecified None	308 (34.0%) 427 (47.1%) 130 (14.3%) 42 (4.6%)
Trastuzumab (*n* = 119) Yes No Unknown	75 (63%) 4 (3.4%) 40 (33.6%)
OFS method (*n* = 660) None GNRHa Surgery/Radiotherapy	603 (91.4%) 46 (7.0%) 11 (1.7%)

n: number; BCS: Breast conserving surgery; OFS: Ovarian function suppression; GNRHa: Gonadotropin-releasing hormone agonist
